# Structural Characterization and In Vitro Antioxidant Activity of Metallothionein from *Oratosquilla oratoria*

**DOI:** 10.3390/molecules27072320

**Published:** 2022-04-03

**Authors:** Guang-Ming Mei, Xiao-Hua Wu, Xiao-Jun Zhang, Jie Gu, Yi Fang, Chun-Ying Meng, Wen-Ge Yang

**Affiliations:** 1 Zhejiang Provincial Key Lab of Mariculture & Enhancement, Zhejiang Marine Fisheries Research Institute, Tiyu Road 28, Zhoushan 316021, China; meigm123@163.com (G.-M.M.); xiaojun3627@163.com (X.-J.Z.); nettsea@163.com (J.G.); fyjhsr@126.com (Y.F.); mengchunying1@126.com (C.-Y.M.); 2 College of Food and Pharmaceutical Sciences, Ningbo University, Qixing South Road 169, Ningbo 315800, China; 3 Zhoushan Hospital Affiliated to Wenzhou Medical College, Dingshen Road 739, Zhoushan 316004, China; xiaoxiaohua113@126.com

**Keywords:** *Oratosquilla oratoria*, metallothionein, extraction, purification, antioxidant activity

## Abstract

We report here the purification of a novel metal-binding protein from *Oratosquilla oratoria* (*O. oratoria* MT-1) by gel and ion-exchange chromatography. SDS-PAGE and MALDI-TOF analyses demonstrated that isolated *O. oratoria* MT-1 was of high purity with a molecular weight of 12.4 kDa. The fluorescence response to SBD-F derivatives revealed that *O. oratoria* MT-1 contained a large number of sulfhydryl groups, which is a general property of metallothioneins. Zn and Cu metal stoichiometries for *O. oratoria* MT-1 were 3.97:1 and 0.55:1, respectively. The proportion of cysteine (Cys) residues in the amino acid composition was 32.69%, and aromatic amino acids were absent. The peptide sequence coverage with *Macrobrachium rosenbergii* calmodulin (accession AOA3S8FSK5) was 60%. Infrared spectroscopy of *O. oratoria* MT-1 revealed two obvious peaks at absorption frequencies for the amide I band and the amide II band. CD spectra revealed that the secondary structure was mainly composed of random coil (57.6%) and β-sheet (39.9%). An evaluation of in vitro antioxidant activity revealed that isolated *O. oratoria* MT-1 has strong reducing activities, exhibiting scavenging rates for DPPH and OH of 77.8% and 75.8%, respectively (IC_50_ values 0.57 mg/mL and 1.1 mg/mL). *O. oratoria* MT-1 may be used as a functional additive in cosmetics, health foods, and medical products, as well as a reference material for quantitative analysis of metallothionein in such products.

## 1. Introduction

Metallothioneins (MTs) are a family of low molecular weight (usually Mw < 10 kDa) Cys-rich (30–33% of total amino acids) proteins that are ubiquitous across many organisms [1,2,3,4,5,6,7]. MTs characteristically demonstrate strong resistance to thermal stress [8,9,10] and protease digestion [1,4]. The number and the location of Cys residues in natural MTs are conserved across many species, and these are organized as repeat arrangements of Cys-X-Cys or Cys-Cys motifs that bind Cu^2+^, Zn^2+^, or Cd^2+^ [11]. Thus, MTs typically exhibit a strong metal binding capacity and a strong redox activity. MTs are primarily involved in the storage, transportation, and metabolism of trace metal elements; additional functions include detoxification and chelation of nonessential heavy metals, regulation of homeostasis, elimination of free radicals, protection of tissues from oxidative damage, and antagonism of ionizing radiation [12,13]. MTs also provide active centers for other metalloproteins and metalloenzymes [14]. MTs have been isolated from vertebrates, invertebrates, plants, and microorganisms, and the physicochemical properties of these isozymes are essentially the same [15]. Potentially useful MTs have also been isolated from several marine organisms, including sponges [16], mussels [17], shrimp [18], crabs [19], sea urchins [20], and fish [21].

Since heavy metal stress is known to induce an increase in MT synthesis in various organisms [8,16,21], MT expression may be used as an early warning indicator to monitor heavy metal pollution in marine environments [9,17,22]. MTs have also been widely applied in health food and medicine, where they are used to maintain normal metabolism in an organism; for example, MTs have been utilized for heavy metal detoxification and antioxidation damage [23,24,25]. These research reports demonstrate that the applications for MT are only increasing. At present, the only commercial MT sold on the Chinese market is Zn-MT. In this case, industrial preparation is via separation and purification after extraction from the liver tissue of Zn-induced rabbits. Due to the complexity of the production process and its high associated costs, the market price of commercialized MT is relatively high (approximately ¥1500/5 mg; purity, 95%), and the products are usually in short supply. This financial impediment greatly limits the practical applications of MT. To achieve cost-effective, practical production of MT and widen its applications, it is essential to find low-cost raw MT materials from which high-purity MT products can be developed.

*O. oratoria* is a perennial benthic crustacean, and this abundant resource is widely distributed around the coastal areas of China, Japan, and the Philippines. In 2014, *O. oratoria* production in China reached 294,000 tons. *O. oratoria* feeds on meiobenthos, organic debris, microorganisms, and thus, heavy metals and other pollutants easily accumulate in its body. According to recent studies, *O. oratoria* has a strong ability to accumulate Cd, Cu, Zn, and other elements within the marine environment [26,27,28]. Since MT content can be induced in organisms under heavy metal stress, *O. oratoria* may be an excellent raw material for preparing MTs. At present, research hotspots concerning MTs are mainly focused on the stress response mechanisms and biological functions of MTs. To date, the reported preparation methods for MTs mostly involve cloning the coding sequence of the MT, inducing an overexpression in bacteria or fungi, and then isolating the protein through a series of purification steps [18,29,30,31]. Only a few studies have centered on the direct extraction of high-purity MTs [32,33,34], and there are no reports on the extraction of MTs from marine crustaceans (including *O. oratoria*). Previously, our team successfully isolated three Zn-binding proteins, MT-M-I and MT-M-II, from metal-exposed hairy clams (*Arca subcrenata*) [32], and F1-2 from *Mizuhopecten yessoensis* [33]. In the present study, a novel MT, *O. oratoria* MT-1. was isolated to high purity from *O. oratoria* for the first time, and its antioxidant activity was studied in vitro. Due to its biological activities in binding heavy metals and scavenging free radicals found in the present work, *O. oratoria MT-1* may be used in cosmetics, health foods, and medical products. It can also be used for the quantitative analysis of MTs and as a quality control for the above products. The production of *O. oratoria* MT-1 is also consistent with the comprehensive utilization of marine crustacean biological resources.

## 2. Results and Discussion

### 2.1. Purification of O. oratoria MT-1

**Figure 1:**
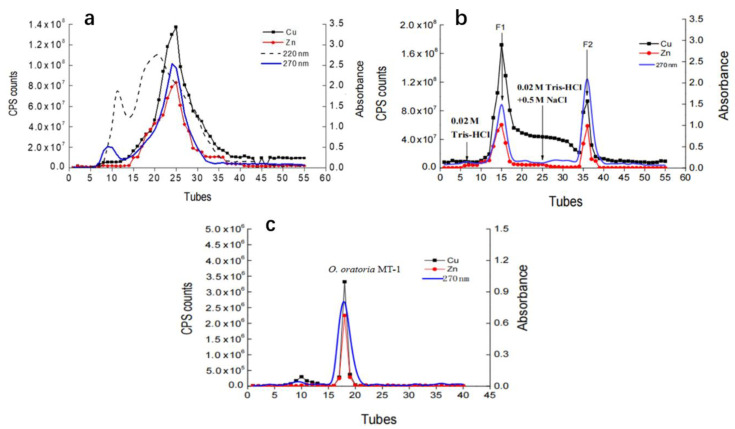
Purification profiles of *O. oratoria* MT-1. (**a**) Separation on Sephadex G-50 gel filtration. Both CPS (count per second) of Cu, Zn in the ICP-MS signal responses, and absorbance at 270 nm and 220 nm were detected in each elution tube. Crude *O. oratoria* MT were obtained by collecting the tubes with both high responses to the above two indicators. (**b**) Further separation of *O. oratoria* MT on the DEAE ion exchange. Two fractions of F1 and F2 were obtained by detection of CPS of Cu, Zn, and absorbance at 270 nm in the elution tubes. (**c**) Desalination on Sephadex G-25 gel filtration. F1 was further refined to obtain *O. oratoria* MT-1.

First, we obtained a crude fraction of the metal-binding proteins using gel filtration. The resulting gel filtration chromatogram on Sephadex G-50 is shown in Figure 1a. The response signals for Cu and Zn were significantly higher than those for Cd, Pb, and As (not shown in the figure due to their low responses). We observed a good correspondence between the ICP-MS signal responses (for Cu and Zn) and UV absorbance (at 270 nm and 220 nm) in the 16th–38th tubes. The collection tubes corresponding to this elution peak were pooled and then lyophilized. Since the UV absorption peak positions of MTs with bound metal are typically 270 m for Cu-MT, 250 nm for Cd-MT, and 220 nm for Zn-MT [35], we inferred that the metal-binding proteins prepared from *O. oratoria* were mainly in the form of Cu-MT and Zn-MT.

In general, the genes encoding MTs express tissue-specific subtypes due to polymorphisms [36]. Since Sephadex G-50 gel column chromatography could not separate the MT subtypes, DEAE Sepharose anion exchange chromatography was used to isolate subtypes from the crude *O. oratoria* metal-binding protein fraction. The elution curve for ion exchange chromatography is shown in Figure 1b. The 11th–20th tubes (peak F1) and the 33rd–40th tubes (peak F2) were separately pooled and subsequently lyophilized.

Next, Sephadex G-25 gel chromatography was used for further purification and desalination of F1 components (this was necessary, because a high-salt buffer was used to elute the sample in the previous step). The elution curve is shown in Figure 1c. The 15th–22nd fractions were combined and freeze-dried. Subsequent structural characterization revealed that purified F1 exhibited the typical properties of an MT, in which a high Cys content, a large number of sulfhydryl groups [2,4,5], a strong metal binding capacity [3,11] and a strong redox activity [12,13] were all observed. The desalted F1 fraction was used as the high purity preparation of *O. oratoria* MT-1. Due to the limited yield (calculated by the obtained amount, the yield of fraction F2 was only 51.2% of F1), we have not prepared enough of fraction F2 for more follow-up structural characterization.

### 2.2. Molecular Weight Determination

**Figure 2:**
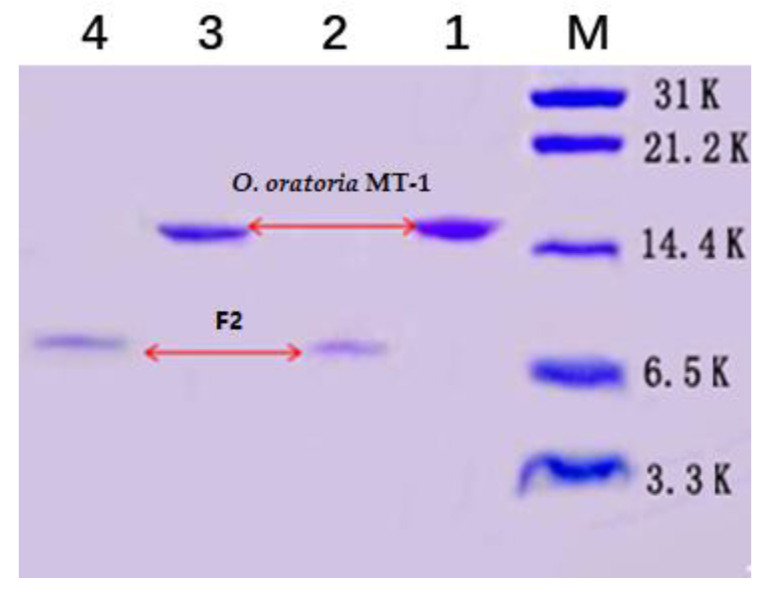
SDS-PAGE analysis of *O. oratoria* MT. M: Marker (Mw 3.3-31 kDa); (1,3) prepared *O. oratoria* MT-1 and (2,4) F2 fraction obtained by DEAE ion exchange chromatography.

SDS-PAGE analysis revealed that *O. oratoria* MT-1 and F2 migrated as single bands (Figure 2), indicating that the two prepared proteins were of high purity. Compared with the electrophoresis bands of marker Mw of *O. oratoria* MT-1 was calculated to be approximately 15 kDa, much higher than the MT expected value (typically Mw < 10 kDa) [4], while the Mw of F2 was about 8 kDa. Although the free sulfhydryl groups in Cys residues of MTs are easily oxidized to form intermolecular disulfide bonds, resulting in molecular polymerization, this may not apply to the present study, because the loading buffer in SDS-PAGE contained a strong reducing agent, β-mercaptoethanol, which can break the disulfide bonds to obtain linear polypeptide chains [37]. However, the metal thiolate clusters in MTs may assist in making the protein structure in a compact form and limiting the loading of SDS on the surface of the protein, which can reduce the amount of negative charges in proteins, affecting their migration on electrophoresis and resulting in a higher apparent Mw [37,38,39,40]. Therefore, a more accurate method was required to calculate the precise Mw for *O. oratoria* MT-1.

**Figure 3:**
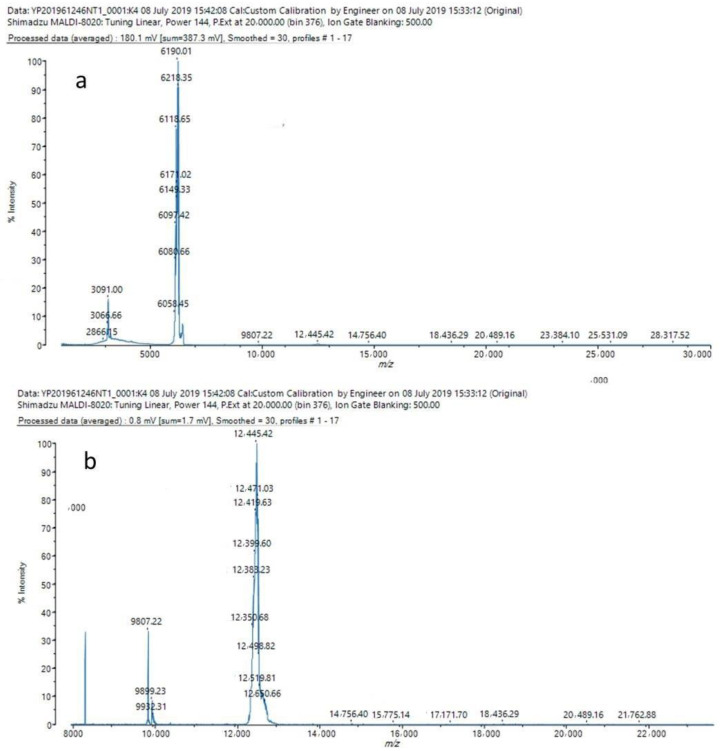
MALDI-TOF spectra of *O. oratoria* MT-1. All mass data were acquired under the following conditions: 3,5-dimethoxy-4-hydroxycinnamic acid as the matrix; a 355-nm N_2_ laser; positive ion linear flight mode; flight tube length 1.22 m; accelerating voltage 20 kV. (**a**) The *m*/*z* ranged 1000~30,000. (**b**) The *m*/*z* ranged 8000~24,000.

MALDI-TOF spectrometric analysis of *O. oratoria* MT-1 in the mass-to-charge ratio (*m*/*z*) range of 500–30,000 is shown in Figure 3a, and Figure 3b demonstrates the regional enlarged view in *m*/*z* 8000–30,000. There were few miscellaneous peaks, indicating that the *O. oratoria* MT-1 preparation was highly pure; *m*/*z* 6190 was the double-charged molecular ion peak [M + 2H]^2+^ for *O. oratoria* MT-1, while *m*/*z* 12,445 was the single-charge molecular ion peak [M + H]^+^. Based on the above results, the Mw of *O. oratoria* MT-1 was calculated as 12.4 kDa. A Zn-MT of similar Mw (14.4 kDa), used as a MT reference in the present work (named Rabbit liver MT-1, with the product batch of 002019120201), was obtained from rabbit liver by Dalian Free Trade Zone Lianhe Botai Biological Technology Co., Ltd. in China. Likewise, we previously reported that the molecular weights of two purified MTs (MT-M-I and MT-M-II) obtained from metal-exposed *Arca subcrenata* were both slightly >17 kDa rather than the expected value (usually <10 kDa) [32]. In addition, a purified Cu-MT isoform from *Sinopotamon henanense* was reported to exhibit an Mw of 13.9 kDa [34]. These diverse observations are consistent with the high Mw of *O. oratoria* MT-1 prepared here. These discrepancies may be explained by species specificity in MTs from marine organisms. 

### 2.3. Fluorescence Derivatization of Sulfhydryl Groups

**Figure 4:**
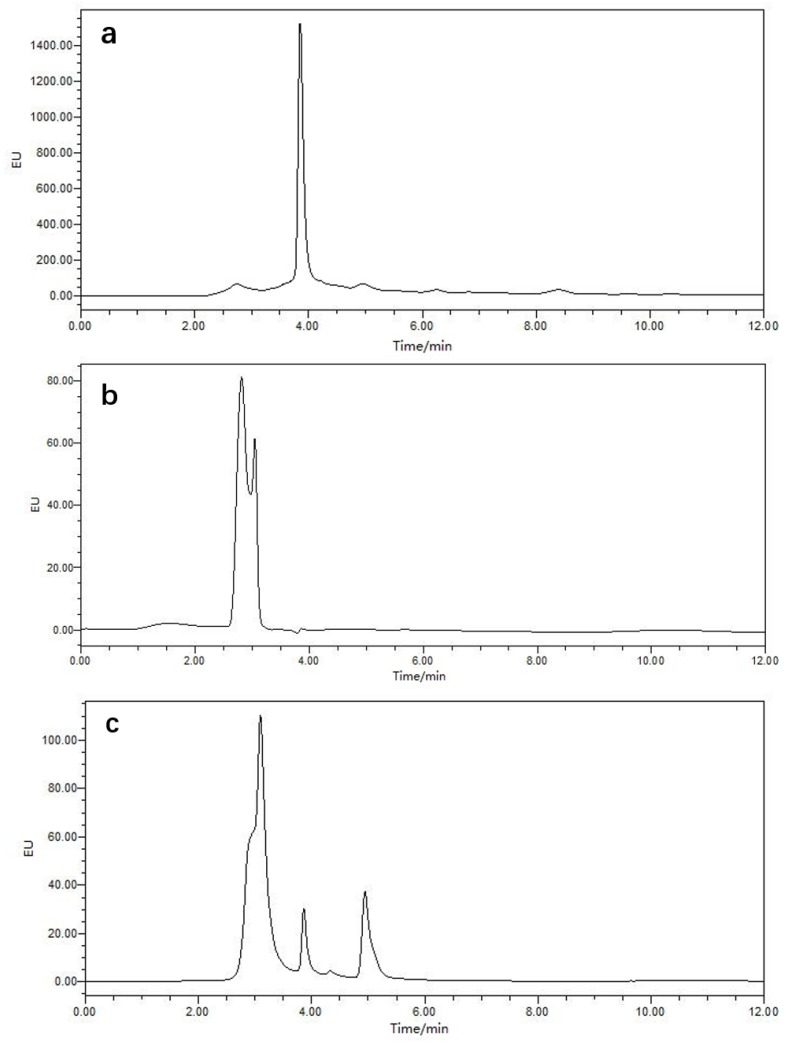
Chromatograms of derivatization between the sulfhydryl group and SBD-F. The analytes were separated by an XBridge C_18_ column and analyzed by high-performance liquid chromatography coupled with a fluorescence detector. (**a**) Chromatogram of *O. oratoria* MT-1 with the complete reaction solvent system (boric acid + EDTA +TCEP + SBD-F). (**b**) Chromatogram of the complete reaction solvent system (boric acid + EDTA +TCEP + SBD-F) itself. (**c**) Chromatogram of *O. oratoria* MT-1 with the solvent system (boric acid + EDTA +TCEP). EU, fluorescence intensity at an excitation wavelength of 380 nm and an emission wavelength of 510 nm. EDTA, ethylene diamine tetra acetic acid. TCEP, Tris(2-carboxyethyl)phosphine. SBD-F, 7-fluorobenzo-2-oxa-1,3-diazole-4-sulfonic acid ammonium salt.

Since MTs have a much higher Cys content than most proteins, an assessment of the sulfhydryl groups would facilitate the development of a highly selective assay whose sensitivity would be enhanced by a reaction with a fluorogenic compound, 7-fluorobenzo-2-oxa-1,3-diazole-4-sulfonic acid ammonium salt (SBD-F) [41,42]. An analysis by high-performance liquid chromatography (HPLC) coupled with a fluorescence detector (FLD) is shown in Figure 4. In the presence of *O. oratoria* MT-1 and the complete reaction solvent system (boric acid + ethylene diamine tetra acetic acid (EDTA) + Tris(2-carboxyethyl)phosphine (TCEP) + SBD-F), a high-intensity chromatogram peak was observed at approximately 4 min (Figure 4a). Chromatograms of the complete reaction solvent system (boric acid + EDTA +TCEP + SBD-F) itself and *O. oratoria* MT-1 with the reaction solvent system (boric acid + EDTA +TCEP) are shown in Figure 4b,c, respectively. Therefore, it can be inferred that a derivatization reaction happened between *O. oratoria* MT-1 and SBD-F and that a large number of sulfhydryl groups were constituent components of the prepared protein. These results are consistent with the general properties of MTs [2,4,5].

### 2.4. Analysis of Metal Stoichiometries

The metal content analyses for a commercially available MT (Rabbit liver MT-1) and purified *O. oratoria* MT-1 are detailed in Table 1. The Zn contents of Rabbit liver MTs and *O. oratoria* MT-1 were highest, followed by the Cu contents. Adjusting for the Mw of each protein, the Zn and Cu metal stoichiometries of Rabbit liver MT-1 were approximately 5.5:1 and 0.5:1. While the Zn stoichiometry of *O. oratoria* MT-1 was lower (3.97:1), the Cu stoichiometry (0.55:1) of *O. oratoria* MT-1 was comparable. These results mirrored the metal contents of *O. oratoria* muscle tissue (Zn > As > Cu; Table 2), indicating that purified *O. oratoria* MT-1 was mainly present in the Zn-MT and Cu-MT forms. In contrast, bound As metal was not detected in *O. oratoria* MT-1, probably reflecting the fact that As in marine organisms exists mainly as small organic–arsenic compounds, such as dimethylarsinic acid, arsenobetaine, arsenocholine, and arsenic sugar [43,44,45]. The Zn stoichiometry (approximately 4:1) of *O. oratoria* MT-1 was lower than that of MT-M-I and MT-M-II from *Arca subcrenata* (6:1 and 7:1) [33] and lower than that of an MT isoform from *Homarus americanus*, which exhibits a coordinating stoichiometry of six Zn^2+^ or Cd^2+^ [46]. The heterologous expressed MT of *Sinopotamon henanense* was observed to bind 15 equivalents of Zn^2+^ in vivo (as analyzed by ESI-TOF-MS), but only six equivalents of Zn^2+^ were required to form the stable metal-S coordination bonds in the tetrahedral structure that coordinates Zn [47]. In most cases, MTs bind 7–12 metal ions [48]. The relatively low Zn stoichiometry of *O. oratoria* MT-1 reported in the present study may be related to the use of ICP-MS for analysis, which has lower precision than ESI-TOF-MS. Conversely, Zn metal may be lost during isolation and purification because of unstable Zn^2+^ binding to *O. oratoria* MT-1.

### 2.5. Amino Acid Composition

The amino acid compositions of *O. oratoria* MT-1 and Rabbit liver MT-1 are shown in Table 3. The Cys content was the highest in *O. oratoria* MT-1, accounting for 32.69% of the total amino acids. The observed Cys content of *O. oratoria* MT-1 was slightly higher than that of Rabbit liver MT-1 (31.99%). Aromatic amino acids (Phe, Trp, and Tyr) were not detected. This is a common feature of proteins in the MT family [4,5], contributing to our confidence in identifying *O. oratoria* MT-1 as an MT.

### 2.6. Peptide Sequence Analysis

**Figure 5:**
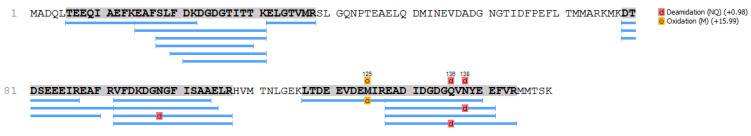
Protein coverage of *O. oratoria* MT-1 and *Macrobrachium rosenbergii* calmodulin. Blue streak represented the matched peptide sequences between the two proteins.

Successful identification of a peptide sequence by high-resolution mass spectrometry is dependent on the information integrity of proteins in the database. Therefore, choosing the appropriate species as a search condition is a key factor. At present, the protein sequence data for *O. oratoria* MT is not available. To expand the scope of our database comparison, crustaceans were selected as the relevant source for the comparison of *O. oratoria* MT-1 peptide sequences. In total, 560 peptides were identified in *O. oratoria* MT-1 after trypsin digestion. One of the highest matched proteins is shown in Table 4, and the details of the matching peptides are shown in Figure 5 and Table 5. The peptide sequence coverage was 60% for *Macrobrachium rosenbergii* calmodulin (accession AOA3S8FSK5). Calmodulins have a highly conserved molecular structure encoded in their protein sequences, and their biological activities are manifold, in addition to participating in various intracellular signal transduction pathways [49]. Since purified *O. oratoria* MT-1 exhibited high homology with calmodulin, it can be inferred that *O. oratoria* MT-1 may also possess calmodulin-like biological activities.

Through an online search of MTs on the UniProt website, it was found that the sequence information for MTs was mainly obtained from mammals, and no crustacean MT sequences were found. Therefore, the identification results for peptide sequence alignment of the prepared *O. oratoria* MT-1 are greatly affected by the limited database (which contains few crustacean proteins). While *O. oratoria* MT-1 and *Macrobrachium rosenbergii* calmodulin shared a high peptide sequence coverage, segments containing Cys residues were missing from the matched peptides. Thus, there is an apparent contradiction with the results of the amino acid composition analysis. However, the principles of the two detection methods are completely different. If the database had included the relevant protein information, the number of matched peptide sequences would be richer and more reliable. Nonetheless, Cys-rich peptides, including AWGSPCEICNPAECDCPIGFAK, CCYDTCLR, and ACNCLLLK, could be matched with other proteins. The matching of the peptide sequences to other proteins from the same or similar species should help us identify possible homologous proteins to *O. oratoria* MT-1, and it should provide information concerning potential biological functions.

### 2.7. Spectroscopic Characteristics of O. oratoria MT-1

#### 2.7.1. IR(Infrared) Spectroscopy

**Figure 6:**
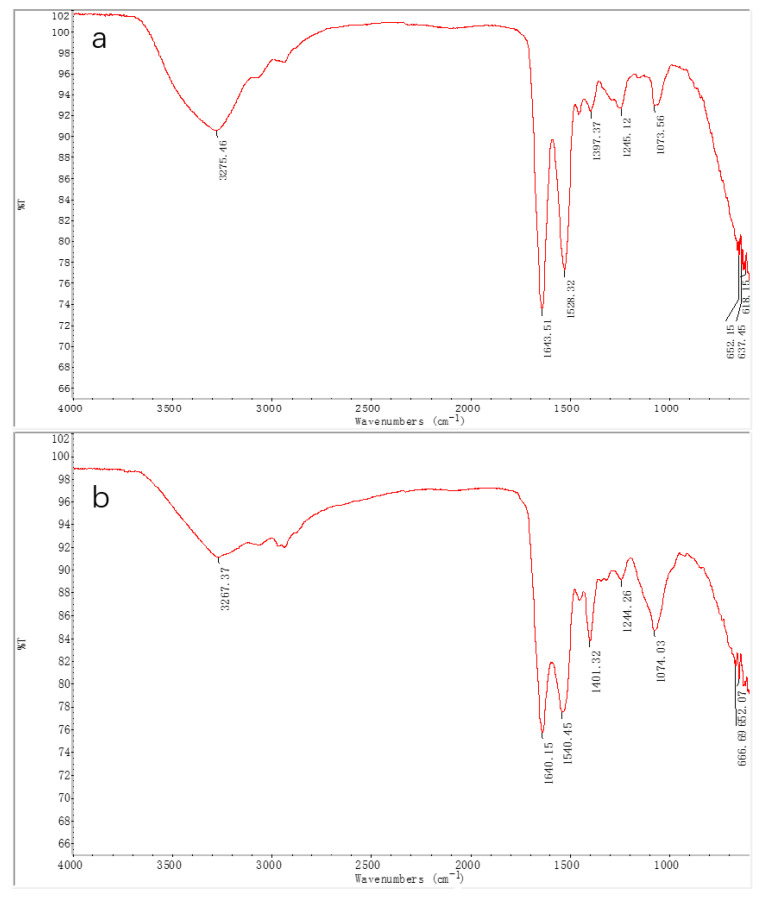
Infrared spectroscopy in the scanning wavenumbers of 400–4000 cm^−1^ with the resolution of 4 cm^−1^. The ordinate axis is the transmittance percentage (% T) of the analytes prepared by mixing MTs and potassium bromide powder at a mass ratio of 1:100 and pressing into tablets. (**a**) Rabbit liver MT-1. (**b**) *O. oratoria* MT-1.

As a rule, protein conformation is determined by regular secondary structures (α-helix, β-sheet, and β-turn) and irregular structures (loops and random coil) [50]. To analyze the *O. oratoria* MT-1 secondary structure, we performed IR spectroscopy. IR spectroscopy is a powerful tool that provides information on protein conformation and structural dynamics [51]. The infrared spectra of three standard amide bands are widely accepted as the diagnostic for specific secondary structure elements of a protein. These standard amide bands are the amide I band around 1700–1600 cm^−1^ (mainly C=O stretch), the amide II band around 1600–1500 cm^−1^ (C=N stretch and N-H bending vibration), and the amide III band around 1330–1220 cm^−1^ (N–H bending and C–N stretching vibration) [52]. In general, absorption band frequencies at 1600–1640 cm^−1^ are diagnostic of β-sheets, absorption band frequencies in the 1640–1650 cm^−1^ range are diagnostic of random coils, absorption band frequencies around 1650–1658 cm^−1^ are diagnostic of α-helices, and absorption band frequencies in the 1660–1695 cm^−1^ range are diagnostics of β-turns [53]. As demonstrated in Figure 6, the mid-infrared spectra (400–4000 cm^−1^) of *O. oratoria* MT-1 exhibited great similarities with Rabbit liver MT-1 (both in absorption frequencies and intensities). Two relatively strong bands at absorption frequencies of 1641 and 1540 cm^−1^ were observed in *O. oratoria* MT-1, representing amide I and amide II, respectively. The peak intensity of the amide III band (1245 cm^−1^) was weak, and a conspicuous band was found at 1074 cm^−1^ (stretching vibration of C–C and C–OH). The amide band I and amide band II of Rabbit liver MT-1 were found at 1644 and 1528 cm^−1^, respectively, and the amide band III was also weak. Furthermore, the infrared spectra of both proteins exhibited a broad soft band around 3270 cm^−1^ (O–H stretching vibration). Together, the IR data provide further confirmation that *O. oratoria* MT-1 is a MT. Furthermore, the characteristic absorption peaks at 1640 cm^−1^ indicate that both *O. oratoria* MT-1 and Rabbit liver MT-1 contain a high percentage of a random coil secondary structure.

#### 2.7.2. CD (Circular Dichroism) Spectroscopy

**Figure 7:**
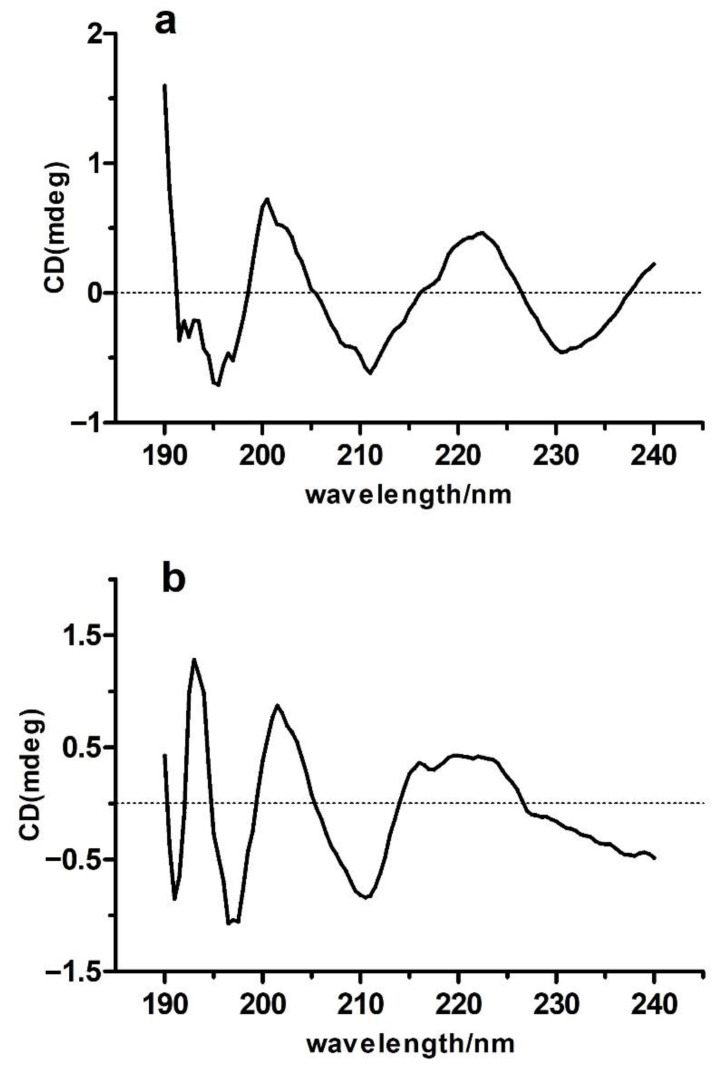
Circular dichroism spectroscopy in the scanning wavelengths of 190–260 nm with a bandwidth of 0.5 nm. The ordinate axis is the molar ellipticity, indicating the analyte’s absorption difference between the left polarized light and the right polarized light. (**a**) Rabbit liver MT-1. (**b**) *O. oratoria* MT-1.

Protein CD spectra provide secondary structure information content by reporting on peptide bond absorption in the far ultraviolet region (185–245 nm). Specifically, an α-helical protein exhibits a positive peak near 192 nm and two negative shoulder peaks at 207–210 nm and 221 to 222 nm. β-sheet-containing proteins exhibit a negative peak at 216–218 nm and a positive peak at 185–200 nm. β-turn-containing proteins exhibit a positive peak around 205 nm, a weak negative peak at 220–230 nm, and a strong negative peak at 180–190 nm. Finally, the presence of a negative peak near 198 nm and a positive peak near 220 nm is characteristic of a random coil structure [54,55]. As shown in Figure 7, the CD spectrum of *O. oratoria* MT-1 exhibited a negative peak at 197.5 nm and a broad positive peak around 220 nm, which are typical characteristics of a random coil structure in proteins. In addition, a strong positive absorption peak at 191 nm and a small reversal peak at 216 nm provide evidence of the β-sheet secondary structure in *O. oratoria* MT-1. Characteristic absorption peaks for β-turn and α-helix were not present. Likewise, random coil and β-sheet were the predominant secondary structures in Rabbit liver MT-1. The proportions of each secondary structure in *O. oratoria* MT-1, as determined by the CD estimation program (Table 6), were 57.6% random coil, 39.9% β-sheet, 2.0% β-turn, and 0.6% α-helix. For comparison, the proportions for Rabbit liver MT-1 were 58.2% random coil, 28.0% β-sheet, 7.3% β-turn, and 6.5% α-helix (Table 6). The total contents of α-helix, β-sheet, and β-turn in *O. oratoria* MT-1 and Rabbit liver in MT-1 were 42.4% and 41.8% (of the total secondary structure), respectively. These results suggest that the two proteins possess a relatively stable conformation.

### 2.8. In Vitro Antioxidant Activity

**Figure 8:**
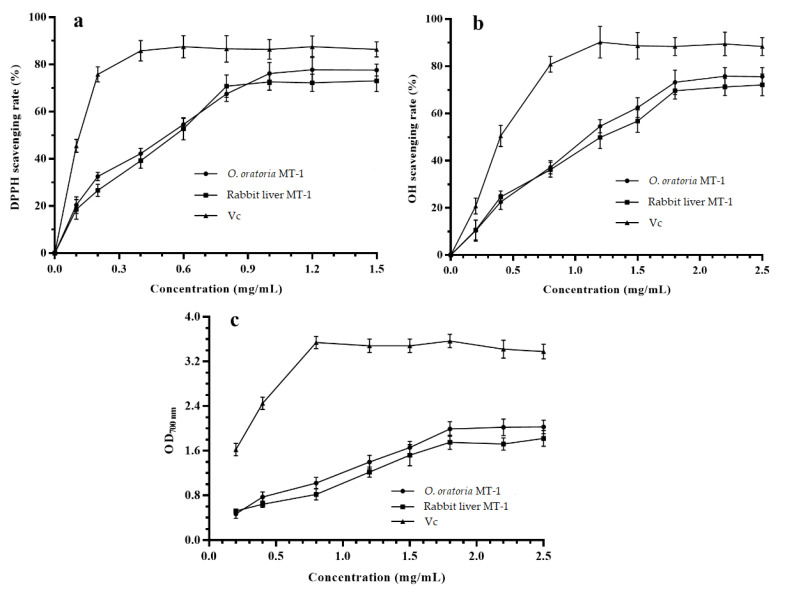
In vitro antioxidant activities of *O. oratoria* MT-1 at different concentrations. (**a**) DPPH radical scavenging capacity. (**b**) OH radical scavenging capacity. (**c**) Reducing power. DPPH: 1,1-diphenyl-2-picrylhydrazyl radical. OH: hydroxyl radical. Vc: Vitamin C. OD_700 nm_: absorbance at 700 nm.

In the presence of antioxidants, the purple color of a DPPH (1,1-diphenyl-2-picrylhydrazyl) solution fades, and this color change can be followed at 517 nm (the maximum absorption peak) [56]. Thus, the antioxidant capacity of a compound can be determined using DPPH absorption. As shown in Figure 8a, the DPPH scavenging rate of Vc (Vitamin C) was stable at 85.8–87.5% over the 0.4–1.5-mg/mL concentration range, with no significant difference between the concentrations (*p* > 0.05). The DPPH scavenging rates of *O. oratoria* MT-1 and Rabbit liver MT-1 increased significantly with a concentration in the 0.1–1.0-mg/mL range. At greater concentrations (1.0–1.5 mg/mL), the DPPH scavenging capacity of both *O. oratoria* MT-1 and Rabbit liver MT-1 were stable, with no significant difference between the concentrations (*p* > 0.05). The highest observed DPPH scavenging rates for *O. oratoria* MT-1 and Rabbit liver MT-1 were 77.8% and 73.1%, respectively. At identical concentrations, the DPPH scavenging rate of *O. oratoria* MT-1 was slightly higher than that of rabbit liver MT-1 (*p* > 0.05) but less than that of Vc (*p* < 0.05). The calculations indicate that the IC_50_ of *O. oratoria* MT-1 activity against DPPH was 0.57 mg/mL, demonstrating that *O. oratoria* MT-1 had good antioxidant capacity.

The OH (hydroxyl) radical is toxic to the human body, where it oxidizes both the protein and DNA and causes damage and necrosis to human cells [57]. As shown in Figure 8b, the hydroxyl radical scavenging rates of *O. oratoria* MT-1 and Rabbit liver MT-1 increased significantly with the protein concentrations in the 0.2–1.8-mg/mL concentration range (*p* < 0.05). In contrast, the hydroxyl radical scavenging rates were relatively stable in the 1.8–2.5-mg/mL concentration range (*p* < 0.05); the highest observed scavenging rates for *O. oratoria* MT-1 and Rabbit liver MT-1 were 75.8% and 72.1%, respectively. The hydroxyl radical scavenging rate of V_C_ also demonstrated a significant dose-dependent relationship over the 0.2–1.2-mg/mL concentration range (*p <* 0.05). At higher V_C_ concentrations, the scavenging rate stabilized; the highest scavenging rate was 90.2%. The IC_50_ values of V_C_, *O. oratoria* MT-1, and Rabbit liver MT-1 were 0.38, 1.1, and 1.2 mg/mL, respectively. While the hydroxyl radical scavenging ability of *O. oratoria* MT-1 was lower than that of Vc, *O. oratoria* MT-1 exhibited good hydroxyl radical scavenging antioxidant ability.

The reducing power of MTs can also be measured by analyzing the protein-dependent Fe^3+^–Fe^2+^ transition. The potential antioxidant activity of a compound is known to mirror its reducing capacity, which has been demonstrated to exert antioxidant action by breaking the free radical chain [58,59]. As shown in Figure 8c, the reducing power of *O. oratoria* MT-1 was slightly higher than that of Rabbit liver MT-1, although both activities were weaker than that of V_C_. The reducing power of *O. oratoria* MT-1 demonstrated a good correlation with the protein concentration in the 0.2–1.8-mg/mL range but was observed to stabilize at higher concentrations.

In our previous work, MT-M-I and MT-M-II prepared from *Arca subcrenata* also exhibited an increase in the DPPH and OH scavenging rates with the protein concentration. The DPPH scavenging rates for MT-M-I and MT-M-II were 85% and 72% (respectively) at a concentration of 100 μg/mL, and the OH scavenging rates were 57% and 60% (respectively) at a concentration of 50 μg/mL [32]. The DPPH scavenging rate for F1-2 prepared from *Mizuhopecten yessoensis* reached nearly 100% at a concentration of 100 μg/mL, while the OH scavenging rate was about 50% at a concentration of 50 μg/mL [33]. The in vitro antioxidant activities of these three MTs (from two different marine shellfish) were higher than that of *O. oratoria* MT-1. This apparent discrepancy can be explained by differences in the materials used and in the purification processes that can cause differences in the molecular structures of the prepared MTs (e.g., copurified inactivated protein). Moreover, the antioxidant activity assay used was different. Nonetheless, the in vitro antioxidant activity study demonstrated that the prepared *O. oratoria* MT-1 had good antioxidant capacity.

## 3. Materials and Methods

### 3.1. Raw Materials

*O. oratoria* was purchased from the Zhoushan International Aquatic Market. All of the *O. oratoria* were caught in the sea area of the Zhoushan fishing ground (29°30′–31°00′ N, 120°30′–125°00′ E). The average body length was 15.5 (±0.5) cm, and the average body weight was 26.2 (±0.4) g. Before processing, surface impurities were washed away with deionized water. During processing, the shell was removed, and the edible parts were harvested and homogenized in a stainless-steel homogenizer (IKA Ultra-Turrax T-25, Staufen, Germany). The homogenates were then stored at −18 °C for future use.

### 3.2. Preparation of O. oratoria MT-1

#### 3.2.1. Buffer Extraction

First, 20-mM Tris-HCl buffer solution (pH 8.0, containing 0.5-mM PMSF and 0.01% β-mercaptoethanol) was added to the sample in a 1:4 ratio. The suspension was stirred at 50 °C for 1 h and then centrifuged at 13,000 r/min for 25 min at 4 °C. The resulting supernatant was heated in a water bath at 80 °C for 3 min (Hitachi CR21G II centrifuge, Tokyo, Japan). The solution was subsequently cooled to room temperature and then centrifuged at 13,000 r/min for 25 min at 4 °C to collect the supernatant. After adding three times the volume of 4 °C precooled ethanol, the total protein was allowed to precipitate at −20 °C overnight. The resulting suspension was then centrifuged at 13,000 r/min for 25 min at 4 °C. Finally, the precipitate was dissolved in 3 mL of Tris-HCl buffer and lyophilized (SJIA-10N-50C, Ningbo Sjia instrument Co., Ltd., Zhejiang, China) to obtain the crude MT extract of *O. oratoria*.

#### 3.2.2. Purification

Gel filtration chromatography. Ten milliliters of 15-mg/mL crude MT solution (dissolved in Tris-HCl) was loaded onto a Sephadex G-50 chromatography column (φ 2.5 cm × 60 cm). The sample was then eluted using 500 mL of 20 mM Tris-HCl buffer (pH 8.0) at a flow rate of 0.8 mL/min. Fractions (8 mL/tube) were collected, and both UV absorbance at 270 nm, 254 nm, and 220 nm (Alpha-1900S spectrophotometer, Shanghai Lab-Spectrum Co.,Ltd., China) and the ICP-MS signal responses (Agilent 7900 ICP-MS, Santa Clara, CA, USA) for Cu, Zn, and other elements were determined.

Ion exchange chromatography. The crude lyophilized powder (120 mg) was dissolved in 8 mL of Tris-HCl and loaded onto a DEAE Sepharose Fast Flow anion exchange column (φ2.5 cm × 40 cm). The sample was washed with 200 mL of 20 mM Tris-HCl (pH 8.0) and then eluted with 200 mL of 20 mM Tris-HCl (pH 8.0, containing 0.5 M NaCl) at a flow rate of 0.8 mL/min. Fractions (8 mL/tube) were collected for further analysis.

Desalination. Ten milligrams (approx.) of the selected fraction was dissolved in 5 mL of ultrapure water and desalinated on a Sephadex G-25 (φ1.5 cm × 60 cm) column. The sample was eluted with 300-mL ultrapure water at a flow rate of 0.8 mL/min and collected in aliquots (8 mL/tube). Finally, selected fractions were lyophilized and stored at −60 °C for future use.

### 3.3. Composition Analysis of O. oratoria MT-1

#### 3.3.1. SDS-PAGE

SDS-PAGE was performed to check the purity of *O. oratoria* MT-1 and to determine its preliminary molecular weight (Mw). To prepare the sample, 100 μL of a 1.0 mg/mL *O. oratoria* MT-1 solution was heated in a water bath at 100 °C for 5 min and then centrifuged at 12,000 rpm for 5 min. Next, 10 µL of supernatant was used for SDS-PAGE electrophoresis analysis under the following conditions [18]: separation gel concentration, 12%; interlayer gel, 10%; spacer gel, 5%; and electrophoresis voltage, first 60 V and then 110 V after the sample had entered the separation gel; electrophoresis was stopped when the bromophenol blue band reached the bottom of the gel. Finally, the gel was recovered, stained for 1 h with 0.1-M Coomassie Brilliant Blue R-250 solution, and then scanned using a GelDoc XR gel imager (Bio-Rad, Hercules, CA, USA).

#### 3.3.2. MALDI-TOF

*O. oratoria* MT-1 solution (1.0 mg/mL) was mixed with the matrix (15 g/L 3,5-dimethoxy-4-hydroxycinnamic acid) at a volume ratio of 2:1 and then incubated at 37 °C for 6 h. Next, 2 μL of the mixed solution was spotted on the ground target plate of a MALDI-TOF MS system equipped with a 355 nm N_2_ laser (Shimadzu MALDI-8020, Kyoto, Japan). All mass spectra were acquired in the 1.0–30.0 kDa range with the following settings: positive ion linear flight mode; flight tube length, 1.22 m; and accelerating voltage, 20 kV.

#### 3.3.3. Derivatization of Sulfhydryl Group

For sulfhydryl group derivatization, 20 μL of *O. oratoria* MT-1 solution (0.5 mg/mL) was mixed with 350 μL of 1 M boric acid (pH 10.5, containing 5 mM EDTA, 10 μL of 300 mM TCEP, and 40 μL of 0.5% SBD-F. The above mixture was then placed in a water bath at 50 °C for 30 min to allow complete derivatization. Next, 50 μL of 4 M HCl was added, and the sample was thoroughly vortexed for 30 s to terminate the reaction. The samples were then filtered through a 0.45 μm filter membrane and analyzed with an HPLC-FLD (Waters 2695 Alliance-2489 FLR detector, Milford, MA, USA) at an excitation wavelength of 380 nm and an emission wavelength of 510 nm. The separation column used was an XBridge C_18_, 5 µm, 4.6 mm × 250 mm (Waters, Milford, MA, USA). An isocratic elution program of 20 mM K_2_HPO_4_ (pH 7.5):acetonitrile:methanol = 80:18:2 and 1.0 mL/min of elution flow rate were applied.

#### 3.3.4. Determination of Bound Metal Element

To determine the bound metal element, 1.0 mg/mL of *O. oratoria* MT-1 aqueous solution was directly injected into an ICP-MS instrument (Agilent 7900 ICP-MS, Santa Clara, CA, USA) for the quantitative analysis of Zn, Cu, Cd, Pb, and As.

#### 3.3.5. Composition Analysis of Amino Acids

To analyze the amino acid composition, 20 mg of *O. oratoria* MT-1 was transferred to a 15-mL glass test tube, and 5 mL of 6 M HCl were added. The test tube was then sealed under nitrogen, and the sample was heated at 110 °C for 22 h to facilitate hydrolysis. After cooling to room temperature, the hydrolysate was transferred to a 10 mL volumetric flask, and the volume was adjusted to 10 mL with pure water. All samples were filtered through a 0.45 μm filter membrane, and the composition of 18 amino acids was analyzed using an automatic amino acid analyzer (Hitachi L-8900, Tokyo, Japan).

#### 3.3.6. Peptide Sequence Analysis

To determine the peptide sequence, 200 μg of *O. oratoria* MT-1 was added to 200 μL of 8 M guanidine hydrochloride (dissolved in 50 mM phosphate solution, pH 8.0) and 5 μL of 1 M dithiothreitol (DTT). The resulting solution was thoroughly mixed and then incubated at 37 °C for 1 h. Next, 10 μL of 1 M iodoacetamide was added, and the sample was incubated at room temperature for 30 min in the dark. To facilitate solvent replacement with 50 mM phosphate solution (pH 8.0), the sample was centrifuged in a 15 mL ultrafiltration tube (molecular weight cut-off, 10 kDa) at 12,000 r/min for 30 min 4 °C. The filter was repeatedly rinsed with phosphate solution to maximize the recovery, and the sample volume was increased to 200 μL. Next, 50 μL of sample solution was digested with 1 μL of 1 mg/mL trypsin (sequencing grade) for 12 h at 37 °C. Finally, 1 μL of 10% FA was added to the sample, and the sample was mixed. Sequence analysis was subsequently performed on an EASY-nLC 1200 (Thermo, Waltham, MA, USA) coupled with a nano ESI Q-Exactive mass spectrometer (Thermo, Waltham, MA, USA). The MS/MS data obtained for *O. oratoria* MT-1 were analyzed and aligned in the NCBI blast database.

### 3.4. Spectroscopy Characteristics

#### 3.4.1. IR Spectroscopy

For IR analysis, 1.0 mg of lyophilized *O. oratoria* MT-1 was mixed with 100 mg of potassium bromide powder and pressed into tablets. The sample was then analyzed using a NICOLET-380 infrared spectrometer (Thermo, Waltham, MA, USA) under the following conditions: scanning spectral range, 400–4000 cm^−1^; resolution, 4 cm^−1^; repeated scanning times, 32; and subtraction background, air.

#### 3.4.2. CD Spectroscopy

For CD analysis, *O. oratoria* MT-1 was first dissolved in distilled water (0.5 mg/mL). The samples were then analyzed at 25 °C using a JASCO-815 spectropolarimeter (JASCO, Tokyo, Japan) under the following conditions: scan speed, 100 nm/min; scan range, 190–260 nm; bandwidth, 0.5 nm; and length of quartz cell, 1.0 cm. CD spectra were corrected for solvent contributions and expressed in terms of specific ellipticities versus wavelength. The proportions of the secondary structure fractions (α-helix, β-sheet, β-turn, and random coil) were determined using the protein secondary structure estimation program CDpro (Jasco, Tokyo, Japan).

### 3.5. In Vitro Antioxidant Activity

#### 3.5.1. DPPH Radical Scavenging Capacity

The DPPH radical scavenging capacity of *O. oratoria* MT-1 was measured according to the method of Huang et al. [60] with a slight modification. A volume of 500 μL of each protein sample at different concentrations was added to 500 μL of 0.1 mM DPPH (in ethanol). The mixtures were shaken and then incubated at 30 °C for 30 min in the dark. The absorbance at 517 nm (A_s_) was then determined using a UV-3200 spectrophotometer (Mapada, Shanghai Meipuda instrument Co., Ltd., China). As a blank control (A_0_), the absorbance of pure water was measured by the same method. Vc (Vitamin C) was used as the reference antioxidant. The DPPH radical scavenging capacity was calculated using the formula: DPPH radical scavenging capacity (%) = (1 − A_s_/A_0_) × 100.

#### 3.5.2. OH Radical Scavenging Capacity

The hydroxyl radical scavenging capacity of *O. oratoria* MT-1 was determined as described by Han et al. [61] with a slight modification. First, 2 mL of protein sample (at different concentrations), 2 mL of 6 mM ferrous sulfate solution, and 2 mL of 6 mM salicylic acid (in ethanol) were added in turn to a 10 mL test tube. After thorough mixing, 2 mL of 6 mM H_2_O_2_ solution was added to initiate the reaction, and the sample was incubated at 37 °C for 30 min in the dark. The absorbance was then evaluated at 510 nm. Vc was used as the reference antioxidant. The hydroxyl radical scavenging capacity was calculated according to the following formula: OH scavenging capacity (%) = [1 − (As − As_0_)/A_0_] × 100, where As represents the absorbance of the protein sample, As_0_ represents the absorbance of the blank control group, and A_0_ represents the absorbance of the protein sample without hydrogen peroxide.

#### 3.5.3. Reducing Power

The reducing power of *O. oratoria* MT-1 was determined as described by Gu et al. [62] with a slight modification. First, 2 mL of protein sample (at different concentrations), 2 mL of 0.2 M PBS (pH 6.6), and 2 mL of 1% potassium ferricyanide solution were added in turn to a test tube. After thorough mixing, the samples were incubated at 50 °C for 30 min. Upon cooling to room temperature, 2 mL of 10% trichloroacetic acid were added. The mixture was then centrifuged at 3000 r/min for 10 min. A 3 mL aliquot of the supernatant was added to 3 mL of pure water and 0.5 mL of 0.1% ferric chloride. After shaking, the mixture was allowed to stand at room temperature for 10 min. Finally, the absorbance was determined at 700 nm. The reducing ability was expressed by the absorbance value, and Vc was used as the reference antioxidant.

### 3.6. Statistical Analysis of Data

Analyses were performed in triplicate, and the results were expressed as the mean ± standard deviation. SPSS 16.0 software (IBM, Armonk, NY, USA) was used for the statistical analyses of the test results. The Duncan multiple comparison method was used for the significance analysis. A *p* < 0.05 indicates a significant difference in the results. Excel 2010 (Microsoft, Redmond, WA, USA) and Origin8.0 software (OriginLab, Northampton, MA, USA) were used to draw and process the charts.

## 4. Conclusions

In the present study, we reported the purification and characterization of *O. oratoria* MT-1, which is a novel metal-binding protein. Structure characterization revealed that *O. oratoria* MT-1 displayed the typical structural features of a metallothionein. Thus, *O. oratoria* MT-1 was present in the Zn-MT and Cu-MT forms, its Cys content was around 32.69%, and the proportions of each secondary structure were random coil (57.6%) > β-sheet (39.9%) > β-turn (2.0%) > α-helix (0.6%). An evaluation of its in vitro antioxidant capacity revealed that *O. oratoria* MT-1 demonstrated strong reducing power and good scavenging capacity for DPPH and hydroxyl radicals. Due to its biological activities in binding heavy metals and scavenging free radicals, *O. oratoria* MT-1 has good application potential as an additive in cosmetics and health food; it can also be used as a reference material for the quantitative analysis of MT in such products.

However, there were also some imperfections in the present work that need to be considered in the future, such as the facts that *O. oratoria* MT-1 was still not fully characterized and that the structural information about the F2 fraction was still unknown. Future studies must consider the accurate protein sequence, tertiary structures, and the binding sites of metal elements in protein molecules. Moreover, animal models to study the antioxidant activities of *O. oratoria* MT-1 or F2 fraction in vivo must be established to provide basic data and theoretical support for their industrial application.

## Figures and Tables

**Table 1:** Metal content analysis in MTs.

Protein Name	Metal Content (in Molar Ratio, mol/mol. Protein) ^a^				
	Cu	Zn	As	Cd	Pb
Rabbit liver MT-1	0.48 ± 0.033	5.56 ± 0.12	ND ^b^	0.00076 ± 0.00002	0.0070 ± 0.0005
*O. oratoria* MT-1	0.55 ± 0.048	3.97 ± 0.09	0.00024 ± 0.00001	0.0013 ± 0.00003	0.0004 ± 0.00002

^a^ The metal content determined by ICP-MS was converted to the molar content of each metal element per mol *O. oratoria* MT-1, according to the atomic mass of each element and the molecular weight of *O. oratoria* MT-1. ^b^ “ND” indicated not detected (value < limit of detection). Data are presented as the means ± SD (*n* = 3).

**Table 2:** Metal content analysis of *O. oratoria* muscle.

Metal Name	Cu	Zn	As	Cd	Pb	Cr
Content ^a^, mg/kg	15.54 ± 2.55	28.45 ± 4.26	16.66 ± 2.92	2.78 ± 0.43	0.072 ± 0.0011	0.28 ± 0.022

**^a^** The metal contents of *O. oratoria* muscles analyzed by ICP-MS are expressed by the milligrams of each metal element per kilogram of fresh muscle tissue. Data are presented as the means ± SD (*n* = 3).

**Table 3:** Amino acid composition analysis.

Amino Acid	*O. oratoria* MT-1		Rabbit Liver MT-1	
	Content ^a^ (g/100 g)	Proportion ^b^ (%)	Content (g/100 g)	Proportion (%)
Cys	32.07 ± 2.23	32.69	31.61 ± 3.24	31.99
Asp	11.48 ± 1.87	11.70	6.36 ± 0.64	6.44
Thr	5.52 ± 0.45	5.63	6.54 ± 0.59	6.62
Ser	5.04 ± 0.62	5.14	14.32 ± 1.68	14.49
Glu	12.16 ± 1.34	12.40	3.11 ± 0.22	3.15
Pro	2.21 ± 0.30	2.25	3.45 ± 0.32	3.49
Gly	5.80 ± 0.37	5.91	6.49 ± 0.55	6.57
Ala	5.70 ± 0.54	5.81	9.81 ± 1.07	9.93
Val	1.51 ± 0.17	1.54	ND ^c^	ND
Met	1.88 ± 0.28	1.92	1.53 ± 0.11	1.55
lle	3.66 ± 0.62	3.73	1.37 ± 0.09	1.39
Lys	10.93 ± 1.01	11.14	14.21 ± 1.22	14.38
His	0.14 ± 0.018	0.14	ND	ND

^a^ The amino acid content in *O. oratoria* MT-1 was determined by an automatic amino acid analyzer, and the results were expressed by the grams of each amino acid per 100 g of *O. oratoria* MT-1. ^b^ Content ratio of each amino acid to the total amino acids. ^c^ “ND” indicated not detected (value < limit of detection). Data are presented as the means ± SD (*n* = 3).

**Table 4:** List of the highly matched protein.

Accession	Coverage (%)	Peptides	Unique	PTM ^a^	Avg. Mass	Description
A0A3S8FSK5	60	18	18	Y	16,866	Calmodulin OS = *Macrobrachium rosenbergii* OX = 79,674 PE = 2 SV = 1

^a^ Post-translational modification. Sequence analysis was performed on a nano ESI Q-Exactive mass spectrometer, and the identified peptide sequences were aligned in the NCBI blast database of crustacean proteins.

**Table 5:** Supporting peptides in the coverage of *O. oratoria* MT-1 and *Macrobrachium rosenbergii* calmodulin.

Peptide ^a^	−10lgP	Mass	Length	ppm	*m*/*z*	RT	Area Sample 1	Start	End
K.EAFSLFDKDGDGTITTK.E	49.53	1843.884	17	1.3	615.6345	49.07	3.02 × 10^7^	15	31
R.VFDKDGNGFISAAELR.H	48.76	1737.869	16	−0.4	869.9412	49.05	7.48 × 10^5^	92	107
L.FDKDGDGTITTK.E	43.86	1296.62	12	−2	649.3159	9.36	9.15 × 10^6^	20	31
F.SLFDKDGDGTITTK.E	39.51	1496.736	14	−1.2	749.3743	27.28	4.82 × 10^7^	18	31
R.EADIDGDGQ(+.98)VNYEEFVR.M	33.82	1955.838	17	7.7	978.934	50.72	0	128	144
K.LTDEEVDEM(+15.99)IR.E	31.76	1364.613	11	−0.9	683.3131	24.88	6.02 × 10^7^	117	127
K.DTDSEEEIREAFR.V	30.14	1595.706	13	−1.2	798.8594	37.23	6.64 × 10^6^	79	91
R.VFDKDGN(+.98)GFISAAELR.H	28.96	1738.853	16	11.6	580.6315	48.98	0	92	107
R.EADIDGDGQVNYE.E	28.76	1423.574	13	−0.4	712.7939	28.32	9.86 × 10^5^	128	140
F.DKDGDGTITTK.E	26.53	1149.551	11	−1.1	575.7823	6.91	3.59 × 10^5^	21	31
R.EADIDGDGQVNYEE.F	25.55	1552.617	14	−0.4	777.3152	29.47	0	128	141
K.EAFSLFDK.D	24.15	955.465	8	−0.3	478.7396	48.62	6.73 × 10^6^	15	22
R.VFDKDGNGFISAA.E	24.11	1339.641	13	−0.4	670.8274	41.29	1.29 × 10^8^	92	104
K.DTDSEEEIR.E	22.51	1092.457	9	−1	547.2352	9.33	1.66 × 10^7^	79	87
R.EADIDGDGQVN(+.98)YEE.F	21.4	1553.601	14	11.5	777.8165	29.45	2.34 × 10^5^	128	141
K.DTDSEEEIREAF.R	20.78	1439.605	12	−0.8	720.8093	44.39	6.85 × 10^6^	79	90
R.VFDKDGNGFISAAE.L	20.11	1468.683	14	−0.9	735.3483	42.11	1.03 × 10^7^	92	105
K.ELGTVMR.S	19.12	804.4164	7	−0.3	403.2153	15.62	0	32	38
L.TEEQIAEFK.E	17.42	1093.529	9	0.5	547.7721	22.94	0	6	14
F.SLFDKDGDGTITT.K	16.04	1368.641	13	−1.5	685.3267	39.09	9.65 × 10^6^	18	30

^a^ The identified peptide sequences were analyzed by high-resolution mass spectrometry (EASY-nLC 1200-nano ESI Q-Exactive mass spectrometer) and aligned in the NCBI blast protein database of crustacean.

**Table 6:** The proportions of the secondary structure in *O. oratoria* MT-1 and Rabbit liver MT-1 (%).

Protein	Secondary Structure ^a^			
	α-Helix	β-Sheet	β-Turn	Random Coil
*O. oratoria* MT-1	0.6	39.9	2.0	57.6
Rabbit liver MT-1	6.5	28.0	7.3	58.2

^a^ The proportions of the four secondary structure fractions (α-helix, β-sheet, β-turn, and random coil) dissolved in distilled water were determined using the protein secondary structure estimation program CDpro.

## Data Availability

All data generated or analysed during this study are included in this published article.

## Sample Availability

Samples of the compounds are available from the authors.
